# The Institutional Library

**Published:** 1914-09-12

**Authors:** 


### THE INSTITUTIONAL LIBRARY.

### A Manual of First Aid.

First Aid in Accidents and Sudden Illnesses. By Duncan Macartney, M.A., M.D., Visiting Surgeon to the Western Infirmary of Glasgow, etc. (Glasgow : St. Andrew's Ambulance Association, 1914.)

This small manual, it is stated in the preface, has been compiled with the aid of Sir George Beatson's larger book, and the first part of it from Professor Cleland's "Elementary Physiology," from which the diagrams are taken with the consent of the publishers. The chapter on transport has been contributed by Dr. Charles Bennett, who has also rearranged the portion devoted to poisons. The preface further states that the rescue work and mining disasters portion has been taken from Mr. Kerr's book on coal-mining. The numerous sketches or illustrations, apart from the photographs, have been supplied by Mr. Gordon Thomas. It will be seen, therefore, that the author disavows any originality for the book as it stands; and it is possible, because it is mainly a compilation, that its practical value may be increased. The type is much too small, and as the number of pages is about the same as that of the British Red Cross Society's First-Aid Manual by Dr. Cantlie, Dr. Macartney would have been well advised to use a larger type. A special feature—and it is a good one—of this manual is the introduction of some excellent photographs and skiagrams, which have apparently been taken under the direction of Dr. D. J. Mackintosh. These photographs, which show methods of treatment for fractures, the application of roller and other bandages, and some practical details, including the compression of some of the principal arteries, together with modes of carrying and helping disabled patients, will enable everyone to understand exactly how to apply the treatment or assistance recommended in each case. No doubt this manual will be used mainly by the members of the St. Andrew's Ambulance Association, for whose assistance it has been published. Some of the sketches as reproduced are very "sketchy," and should a second edition be called for, no doubt the shortcomings we have indicated will be removed.

Florence Nightingale to Her Nurses : A Selection from Miss Nightingale's Addresses to Probationers and Nurses of the Nightingale School at St. Thomas's Hospital. (Macmillan and Co., Ltd. Price 1s. net.)

These addresses bring home to the reader in a startling way how widely separated Florence Nightingale is from some successors in her ideas of what nursing and the true nurse should be. Her ideal has become too often the commonplace gloss on what is a profession, like any other profession, and were her name not associated with these ideals it is even possible that many would repudiate them, in the same way as schoolboys repudiate the Sunday talks of their teachers. Yet the hospital administrator has only to turn over these brief pages—the style of which, as we are prewarned in the Preface, is aimed at those for whom it was necessary "to write very simply, and without too great severity and concentration"—to realise that the practical efficiency of his staff will depend upon the presence or absence of the spirit here inculcated. "A woman," writes Miss Nightingale, "who takes a sentimental view of nursing (which she calls 'ministering,' as if she were an angel) is, of course, worse than useless." Again, how true is still her warning, "Must I tell you again, what I have had to tell you before, that we have a great name in the world for—conceit?" The book, in short, is full of good things, which hospital men would do well to avoid supposing beneath their notice. Let us take this one as a last example : "Believe me, who have seen a good deal of the world, we may give you an institution to learn in, but it is you must furnish the 'heroic' feeling of doing your duty, doing your best, without which no institution is safe, without which training schools are meat without salt."

### A Book for the General Practitioner.

The Newer Physiology in Surgical and General Practice. By A. Rendle Short, M.D., F.R.C.S. Third Edition, Revised and Enlarged. Bristol : John Wright and Sons, Ltd. 1914. Price 5s.

The favourable impression which the first edition of this book created for itself in medical circles less than three years ago has been amply justified by the continued demand for the work. If such has been the verdict on the previous editions, the author need have no apprehension concerning the success of this enlarged and greatly revised edition. Although the problems dealt with in this volume are mostly those connected with surgery, the interest of the chapters is far from limited to the point of view of operating surgeons. It may, indeed, be doubted if there be any other book of similar calibre which appeals so thoroughly to the general practitioner. In it he will find that much of the knowledge he once learnt has been drastically readjusted, and that much has been thrown overboard; but, on the other hand, he will find many new facts and many a wrinkle which he can apply to his practice with benefit to his patients and kudos to himself. It is a thankless task to pull down strongholds of belief; but it is necessary, if only to direct more attention to the true means of giving relief and to avoid the employment of inert and expensive drugs which have a reputation founded upon unsubstantiated impressions. Of the more practical examples of such beliefs we may draw attention to the author's remarks on cutaneous anæsthetics applied to the unbroken skin and to the researches on nutrient enemata. In this edition new chapters have been introduced on vitamines and the genital glands; the articles on surgical shock, the digestive apparatus, the pituitary and pineal glands, and on chloroform poisoning have been largely rewritten. There are also important additions to the chapters on the thyroid and the spinal cord. A practical point which deserves attention is the efficacy of thyroid extract as a substitute for potassium iodide in the treatment of gummata and atheroma. Of the multitude of text-books which the general practitioner is importuned to buy, he may at least regard this volume as a safe investment.

1914, A War Poem. By F. Barber Wells, M.B. Printed and published for the author by W. H. and L. Collingridge, 148 and 149 Aldersgate Street, E.C. The Roll of the Drum (similar title page).

Among the crop of verse excited by the war, the above two poems, by a medical man, have reached us. So far as we know, if the Laureate's recent contribution on the subject be excepted, Dr. Barber Wells is the first medical man to join the versifiers on this occasion. The verses themselves are not technically striking, but as they are sold for the benefit of the Prince of Wales's National Relief Fund, it may be assumed that a fervent patriotic spirit inspired them, and, as verse in that vein, they should be popular.

